# Preliminary Report: Osteoarthritis and Rheumatoid Arthritis Synovial Fluid Increased Osteoclastogenesis *In Vitro* by Monocyte Differentiation Pathway Regulating Cytokines

**DOI:** 10.1155/2022/2606916

**Published:** 2022-05-31

**Authors:** Jani Luukkonen, Johanna Huhtakangas, Sanna Palosaari, Juha Tuukkanen, Olli Vuolteenaho, Petri Lehenkari

**Affiliations:** ^1^Department of Anatomy and Cell Biology, Cancer Research and Translational Medicine Research Unit, Medical Research Center Oulu, Faculty of Medicine, University of Oulu, Aapistie 5, Oulu 90014, Finland; ^2^Rheumatology Unit, Oulu University Hospital, Medical Research Center Oulu, Oulu, Finland; ^3^Department of Clinical Chemistry, Cancer Research and Translational Medicine Research Unit, Faculty of Medicine, University of Oulu, Aapistie 5, 90014 Oulu, Finland; ^4^NordLab Oulu, Kiviharjuntie 11, 90220 Oulu, Finland; ^5^Department of Surgery, Medical Research Center Oulu, University of Oulu and University of Oulu Hospital, Finland

## Abstract

**Background:**

Rheumatoid arthritis (RA) and osteoarthritis (OA) are common joint diseases associated with changes in local, as well as systemic bone structure and osteoclast function. We investigated how the different soluble inflammatory stimuli in these diseases can affect osteoclastogenesis and bone resorption *in vitro. Methods*. Human peripheral blood mononuclear cell-derived osteoclasts were cultured on bone slices with serum from treatment-naïve RA patients and healthy controls and with synovial fluid samples acquired from RA and OA patients. The concentrations of 29 different cytokines and related proteins, including RANKL and OPG, were analyzed in the fluids tested.

**Results:**

RA serum and synovial fluid increased both osteoclastogenesis and bone resorption. Osteoclastogenesis and activity increased more in the cultures containing OA than RA synovial fluid. The osteoclasts cultured in different culture media exhibited different phenotypes, especially the cells cultured with OA synovial fluid were generally larger and had more nuclei. A general increase in proinflammatory cytokines in RA synovial fluid and serum was found. Surprisingly, OA synovial fluid showed lower levels of osteoclastogenesis inhibiting cytokines, such as IL-4 and IL-10, than RA synovial fluid, which at least partly explains more pronounced osteoclastogenesis. No significant difference was found in RANKL or OPG levels.

**Conclusion:**

The proinflammatory stimulus in OA and RA drives the monocyte differentiation towards inflammatory osteoclastogenesis and altered osteoclast phenotype.

## 1. Background

Rheumatoid arthritis (RA) is a chronic autoimmune inflammatory joint disease that targets mainly the synovial tissue, but the exact disease etiology and pathogenesis are unknown [1]. The synovial tissue inflammation is a very common feature of the disease and leads to secondary damage in both the cartilage and bone [1]. The local inflammation is believed to increase osteoclast activity and result in local bone loss specific for the disease [2–4]. In RA, the bone erosions are located at a special milieu at the sides of the joint where the synovial lining is adjacent to the bone and the bone is not covered by articular cartilage. RA is also associated with secondary osteoporosis, which is thought to be a result of generalized inflammatory stimulus that originates from generalized inflammation of the affected joints [5]. The features of excess bone resorption suggest that there are multiple soluble and insoluble signaling molecules that contribute to the excess bone resorption in RA.

Besides RA, osteoarthritis (OA) is another even more common joint-decaying disease, which also results in changes in local bone turnover. OA is a disease of the whole joint associated with mechanical wear of the joint surfaces and involvement of the surrounding tissue, subchondral bone, cartilage, and synovium. OA includes a component of low-grade inflammation (reviewed in [6]) and also complement activation has been suggested to take a part in the pathogenesis [7]. Even in early OA, a local inflammatory component can be seen, and in late stages, this inflammatory reaction increases causing symptoms and pathological changes in the bone metabolism [8]. In OA, increased osteoclast activity is detected in the subchondral bone which leads to structural damages called the herniation pit. Changes can also be found on the border of the cartilage and synovium, similar to RA. Interestingly, in OA joints, sclerosis and ectopic osteophytic bone formation also takes place at the same sites as increased osteoclast activity [9]. Systemic effects on bone are less frequently seen in OA and secondary osteoporosis is not a common feature, probably due to lower levels of systemic inflammatory cytokines [5].

**Figure 1:**
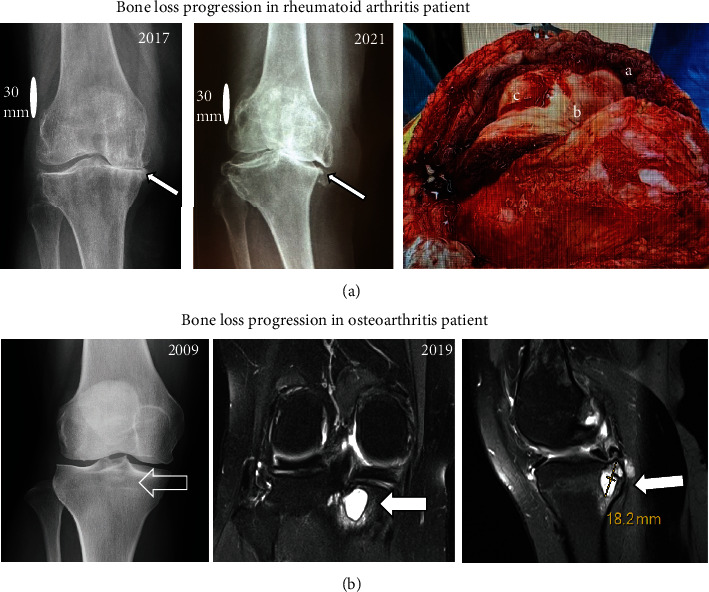
Clinical changes seen in RA and OA patient knees. Panel (a) shows the progression of erosive arthritis due to RA in a knee of female patient during 2017 to 2021 and intraoperative picture during a knee prosthesis operation demonstrates synovial inflammation and hypertrophy (a) along with cartilage (b) and bone damage (c) associated with synovial hypervascularity and oedema. The medial tibial plateau (arrows) is heavily eroded during the follow-up time period. Panel (b) shows X-ray and MRI images of a young female's knees. At the age of 27, only some early OA changes are seen. The magnetic resonance images ten years later show progression of OA, with large subchondral bone loss and a cyst in the proximal tibia as an indication of increased bone degradation.

Local changes seen in a RA and OA patient knees are shown in Figure 1. In RA, the rapid progress of joint wear and deformation due to inflammation is called secondary erosive arthritis. In primary OA, increased bone degradation can often be seen as herniation pits or degenerative cysts. Synovial inflammation with cartilage and bone degeneration can clearly be seen in patients undergoing knee surgery.

The key players contributing to bone changes in both RA and OA are osteoclasts, which are generally described as multinucleated tartrate-resistant acid phosphatase, TRAcP, staining positive giant cells capable of resorbing bone [10] in suitable milieu. In *in vivo,* osteoclastogenesis is induced from mononuclear osteoclast precursors by the macrophage colony-stimulating factor (M-CSF) and receptor activator of nuclear factor kappa-B ligand (RANKL) [11–13]. Osteoclasts can be generated *in vitro* by stimulating peripheral blood-derived mononuclear cells with RANKL and M-CSF [14], and it has been shown that osteoclastic bone resorption can be affected by a multitude of different cytokines, e.g., IL-6 and TNF-*α* increase and osteoprotegerin (OPG) decreases it [11–13]. Spontaneous osteoclastogenesis has been described in cell cultures using human samples, from patients with inflammatory diseases, such as RA synovial tissue and mononuclear cells [2, 3]. This suggests that osteoclastogenesis *in vivo* is a complex, balanced process that is influenced by multiple cytokines and this environment is not easily reproducible in *in vitro* osteoclastogenesis assays.

The size of the osteoclasts has been associated with their activity and larger osteoclasts are more often involved in pathological bone resorption [15–17]. In RA, inflammatory cells, such as macrophages and lymphocytes, present in the inflamed synovial tissue, produce both soluble inflammatory cytokines and insoluble membrane-bound signaling molecules, which increase the activity of residing osteoclasts but also are likely to increase osteoclastogenesis in a form of inflammatory osteoclastogenesis [2]. During inflammatory or inflammation-enhanced osteoclastogenesis, various cytokines produced by these inflammation regulating cells either inhibit or increase the differentiation and activation of osteoclast precursors and osteoclasts. There are also observations suggesting that similar to macrophages, there could be a distinguished phenotype of “inflammatory osteoclasts” that differ from normal osteoclasts, i.e., the inflammatory osteoclasts are smaller and contain fewer nuclei, presenting a more macrophage-like phenotype [2, 18].

Our aim was to create a model to study the complexity of the local and systemic inflammatory stimulus on osteoclastogenesis *in vitro*. Human serum from novel untreated RA patients and synovial fluid (SF) from patients undergoing knee prosthesis operation, as well as human peripheral blood-derived mononuclear cells, were used to produce a controllable osteoclastogenesis model and bone resorption activity model. These approaches allowed us to better understand the pathophysiology of local and systemic bone loss in RA and OA at the cellular level and to detect possible key cytokines that could partially explain the different clinical features of the diseases.

## 2. Methods

### 2.1. Synovial Fluid, Serum, and Cell Acquisition

RA serum, that was used in the osteoclast cultures, was collected from six untreated RA patients at the time of initial diagnosis. At this time point, the patients did not show signs for advanced knee osteoarthritis, and they were not undergoing knee surgery. There is no systematic radiographic data of the possible lower grade KL classification changes in knee or other joints. The RA patients were diagnosed according to the ACR/EULAR 2010 criteria for RA by a rheumatologist, and they were seropositive (mean anti − CCP 232.2 ± 147.9; RF [Rheumatoid factor] 142.4 ± 74.4). Healthy control serum was collected from nine healthy volunteers.

Synovial fluid was acquired from ten RA and OA patients undergoing total knee replacement operations. These patients were all different from those, from whom we could collect serum samples, as described above. All patients in this group had advanced osteoarthritis at least in their knee joints, and the mean Kellgren-Lawrence score for the operated knees was three in both RA and OA groups. All RA patients were seropositive (mean anti − CCP 145.1 ± 121.6; RF 158.6 ± 131.0). RA patients, who the synovial fluids were collected, were treated with various different RA medications that had been changed numerously over the years. Patient data is shown in Table 1. The samples were centrifuged at 2000 g for 10 minutes at 4°C to remove any possible cells and other undissolved material. The supernatant was stored at -80°C. For use in cell cultures, three synovial fluid and serum samples from each group were selected blindly.

Due to low volume of synovial fluid available and the known high variation of cytokines in synovial fluid, the blindly selected synovial fluid samples were pooled to create a standardized disease environment.

Peripheral blood mononuclear cells were isolated from whole blood of a healthy volunteer male donor using the Ficoll-Paque density gradient centrifugation method (GE Healthcare) according to the manufacturer's instructions. Blood samples were collected into heparin-coated tubes to prevent coagulation. After acquisition, the fresh blood samples were diluted 1 : 1 in PBS and layered on top of the Ficoll-Paque solution and centrifuged 400 g, at room temperature for 35 min without brake. Buffy coat was collected and twice suspended to 50 ml PBS and centrifuged 170 g for 10 min to remove platelets. After separation, the mononuclear cell fraction was collected and used immediately for cell culture. The cells were not frozen during the procedure.

No additional trauma was caused during sample acquisition. The patients gave a written informed consent for the use of their samples. The protocol followed the Helsinki Declaration principles in full, and the Northern Ostrobothnia Hospital District Ethical Committee gave an approval for the study and tissue collection.

### 2.2. Osteoclast Cultures Generated with RA and Control Patient Serum

Peripheral blood mononuclear cells were cultured on top of bovine bone slices in 96-well plates (300 000 cells/well, 15 wells/tested serum sample) and differentiated into osteoclasts in alpha-MEM including 100 iU/ml penicillin and 100 *μ*g/ml streptomycin with 20% concentration of tested serum, and additional RANKL 20 ng/ml, M-CSF 10 ng/ml, and heparin 5000 IU/ml 0.20 ml/ml. Three RA and three healthy control sera (one male and two females in each) were used for these experiments. Fetal bovine serum (FBS) was used as a reference serum, and negative control cells were cultured with FBS without added RANKL and M-CSF. The cells were cultured for 14 days, with half of the medium replaced every 3-4 days. The cells were fixed with PFA.

The bone slices were cut from long bovine bones' cortical areas using a diamond saw into 100 *μ*m thin 6 mm round slices and stored in 70% ethanol. Before use, they were rinsed thoroughly in PBS. The 6 mm round slices fit 96-well plate dishes' wells precisely to provide a good baseplate for cell cultures and cells can be fixed directly on them.

### 2.3. Osteoclast Cultures Generated with Synovial Fluid

Peripheral blood mononuclear cells were cultured and differentiated into osteoclasts with the same protocol as for the RA serum test (300 000 cells/well, 15 wells/tested sample), but this time, the serum was replaced with the pooled RA or OA synovial fluid. The pooled synovial fluid was added to the cell culture media as 20% concentration with the addition of 10% healthy human serum. As a control, a culture with 10% healthy human serum was conducted at the same time. The mononuclear cells for the synovial fluid and serum assay were collected from the same donor, but at different times to keep the amount of donated blood in minimum safe levels. In this study, synovial fluid from osteoarthritis (OA) patients was used as a control due to ethical and volume limitations for acquiring synovial fluid from healthy individuals. Healthy control knees have been suggested to contain 6.7 ± 2.3 ml SF (e.g., [19]), but only very little is practically collectable.

### 2.4. Cytokine Assay

Twenty-seven different cytokine and related protein concentrations (IL-1-beta, IL-1 receptor antagonist (IL-1ra)), IL-2, IL-4, IL-5, IL-6, IL-7, IL-8, IL-9, IL-10, IL-12 (p70), IL-13, IL-15, IL-17, eotaxin, basic fibroblast growth factor (FGF), granulocyte colony-stimulating factor (G-CSF), granulocyte-macrophage colony-stimulating factor (GM-CSf), IFN-*γ*, IP-10, MCP-1, MIP-1-alpha, MIP-1-beta, platelet-derived growth factor (PDGF-BB), RANTES, TNF-*α*, and VEGF were analyzed using the Bio-Rad Bio-Plex Pro™ Human Cytokine 27-plex assay, Luminex MagPix Instrument and Luminex xPotent Software from the synovial fluids (10 RA and OA), and serums (6 RA and 9 healthy control). Three samples from each group were used for osteoclast culture assays. The serum samples were diluted two-fold and the synovial fluid samples four-fold. All samples were tested in duplicate.

Synovial fluids were tested also for RANKL and OPG concentrations using an Invitrogen™ eBioscience™ ProcartaPlex Human RANKL Simplex Kit and a TNFRSF11B Human ProcartaPlex™ Simplex Kit for OPG. The test was done to avoid confounding by RANKL and OPG, by showing that in the cell cultures the RANKL concentration used is supraphysiological and the RANKL already present in the samples does not affect osteoclast differentiation in the experiment.

### 2.5. Analysis of Osteoclast Differentiation and Bone Resorption

The fixed cells were stained with a TRAP kit (Sigma-Aldrich) and Hoechst nuclei stain. TRAP positive cells with two or more nuclei were counted as osteoclasts. The osteoclast number in the serum assay was counted using a light microscope from the whole 6 mm diameter bone slice. For the synovial fluid assay, the number of osteoclasts was counted from five random locations on the bone slice under 20x magnification. The number of osteoclasts between the two assays is not directly comparable even though they are from the same donor, since the cells have been extracted from the blood at different time points and different biological factors like cytokine levels in the blood could affect the outcome. This was done to limit the volume of donated blood at a single time to a minimum. Stromal cell number, intensity of TRAP staining, and number of nuclei within osteoclasts were quantified blindly and individually by two researchers under a light microscope from each bone slice. The intensity of TRAP stain and the presence of stromal cells were quantified on a 0-3 nominal scale. Since osteoclast size has been associated with their activity, the average number of nuclei in an osteoclast, which also represents the cell size, was counted on a scale of 0, 2-3 (small), 4-7 (medium), or >8 (large). Additionally, the cells were visualized using a Zeiss LSM 780 confocal microscope.

To visualize and confirm resorption pits on the bone slices before laser microscopy, the cells were brushed off from the slices using a small plastic-tipped brush, and the pits were stained with horseradish peroxidase-conjugated WGA-lectin antibody and DAB stain. The area and volume of resorption pits on a bone slice were measured using an Olympus LEXT OLS4100 laser microscope and software. A bone slice was divided into five sectors and a random area (0.422mm^2^) from each sector was captured and analyzed with 20x magnification. The area and volume of resorption were measured from each captured area. The average depths of the three deepest pits in the area were measured independently.

All statistical analyses were done with the IBM-SPSS 24 program. A nonparametric Mann–Whitney *U* test was used to calculate possible significant differences. Correlation between parameters was assessed using Spearman's rank correlation. *p* values under 0.05 were considered statistically significant.

## 3. Results

### 3.1. Cytokine Assay

**Figure 2:**
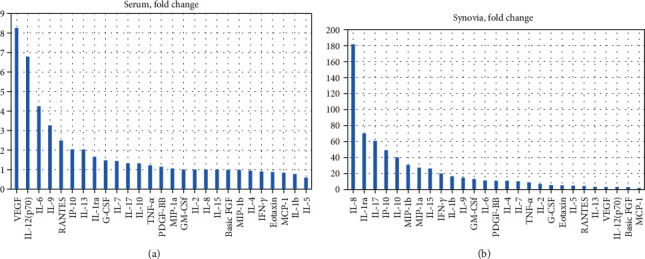
Fold changes of the mean cytokine concentrations between RA and control sample used for cell exposures. Panel (a) shows the fold change of mean cytokine concentration between the healthy controls and the treatment-naïve RA patient serums. Panel (b) shows the fold change of the mean cytokine concentrations between OA and RA synovial fluid gathered at the time of prosthesis operation.

Patient samples used for cell exposures were analyzed for 27 different inflammation-related cytokines to analyze the state of inflammation present in the samples (supplementary file S1). Each sample was analyzed individually as a duplicate, and mean concentrations were calculated for each group. When comparing novel untreated RA patient serum, which was collected at the time of diagnosis, with healthy control serum elevated levels of cytokines were found in RA sera (Table 2 and Figure 2(a)). VEGF was increased 8-fold, IL-12 (p70) 7-fold, IL-6 4-fold and IL-9 3-fold. Other cytokines were elevated less than 2-fold or were not elevated in the RA sera

In synovial fluids, all cytokine levels except PDGF-BB were higher than in the sera as expected, validating the results of our cytokine analyses. All cytokines were elevated in RA SF compared to OA SF. The largest increases were seen in IL-8 (182-fold), IL-1ra (70-fold), IL-17 (59-fold), IP-10 (49-fold), IL-1b (39-fold), MIP-1a (31-fold), MIP-1b (27-fold), IFN gamma (20-fold), IL-2 and IL-9 (15-fold), GM-CSf (13-fold), IL-6 (11-fold), PDGF-BB and IL-4 (11-fold), IL-7 (10-fold), IL-10 and TNF-alpha (9-fold), and other cytokines 5-fold or less (Table 2 and Figure 2(b)).

Since it has been suggested that RF has a confounding effect in multiplexing immunological cytokine analyses [20], we tested the correlation of cytokines with RF levels with a larger set of samples. All serum and SF cytokine results are presented in Supplementary file S1. In serum samples, we found negative correlation between MCP1 and RF (*ρ* = −0.894, *p* = 0.041); and in synovial fluid samples, we found positive correlation between TNF-alpha (*ρ* = 0.768, *p* = 0.044), IL-4 (*ρ* = 0.768, *p* = 0.044), IL-10 (*ρ* = 0.768, *p* = 0.044), G-CSF (*ρ* = 0.808, *p* = 0.028), IL-17 (*ρ* = 0.768, *p* = 0.044), and RF that could indicate either RF interference in the analyses or RF relation to the disease activity. Due to the possible RF interference, these should be interpreted with caution.

Altogether, the cytokines were roughly ten times more concentrated in the synovial fluids, when compared to the serums. In supplementary file S2, Spearman's correlations between all cytokines within each group are shown to elucidate the interconnecting cytokine networks. Strong correlations between different cytokines were seen especially in RA synovial fluids.

There was no difference in OPG concentration in the synovial fluid of OA and RA patients. However, OPG showed statistically significant positive correlation with the KL score (*ρ* = 0.497, *p* = 0.042) in all analyzed SF samples (*n* = 20, Supplementary file S1). Other cytokines did not show correlation with KL score. All, but a single RA RANKL measurement, fell under the detection limit of 7.04 pg/ml. However, as in all cell cultures, RANKL was used in a supraphysiological 20 ng/ml concentration, so the differences seen in osteoclast differentiation are most likely the results of cytokines other than RANKL in synovial fluid. The RANKL concentrations in RA serum are known to be under 1 ng/ml [21].

### 3.2. Microscopy Analysis of Osteoclast Morphology

**Figure 3:**
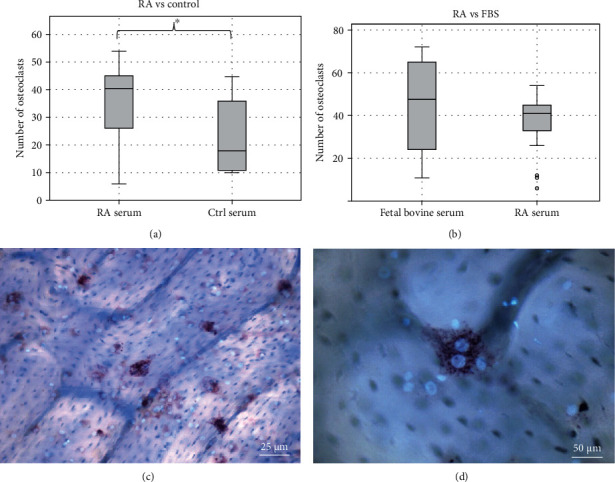
Osteoclast numbers in cultures with RA and healthy serum. Panel (a) shows the mean number of osteoclasts on bone slices in cell cultures with treatment-naïve RA patient serum (*n* = 15) and healthy control serum (*n* = 13). RA serum increased the number of osteoclasts significantly compared to healthy serum. Only multinucleated TRAcP positive cells were counted as osteoclasts. (b) Number of osteoclasts in cultures with FBS, which was used as a positive control. (c) 10x magnification and (d) 20x magnification from RA serum sample. No morphological differences were seen between the cells cultured with RA serum or healthy control serum. Red color indicates TRAcP and blue Hoechst stain marks the nuclei.

The osteoclastogenesis of mononuclear cells grown in different experimental conditions was first analyzed by light microscopy. The numbers of osteoclasts in the serum assay are presented in Figure 3(a). Light microscope images of osteoclasts are shown in Figures 3(c) and 3(d). Single nucleated TRAcP positive cells were not counted as real osteoclasts. RA serum increased the number of osteoclasts significantly compared to healthy serum (mean 36.4 ± SD 15.2 vs. 22.9 ± 13.1). Two healthy control slices were excluded due to technical issues in the samples (i.e., unattached cells or low cell count). The number of osteoclasts was the same in cultures with RA serum and FBS, the variation of osteoclast number was greater in samples with FBS (supplementary figure S3). FBS was used as a reference serum, as it is known from previous studies that it can be used in osteoclast cultures to achieve good results. Due to low number of fetal bovine serum samples analyzed to save cells for the studied human serums, no further conclusions can be done on its effect on osteoclastogenesis and function. No morphological cell differences were noted between different serum cultures. No osteoclastogenesis was seen in negative control sample cultures without RANKL and M-CSF.

**Figure 4:**
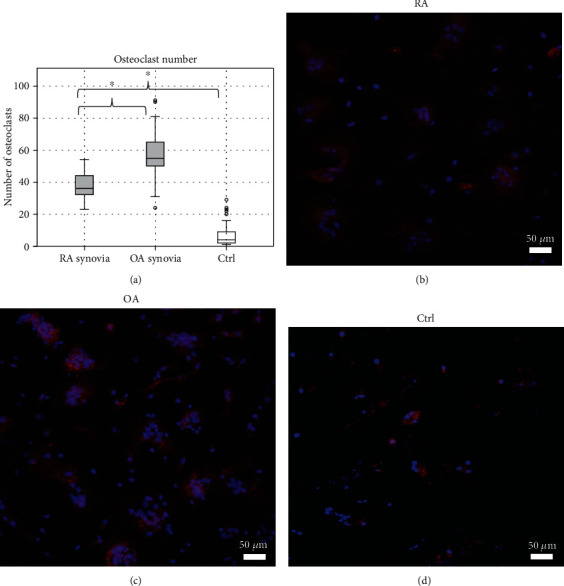
Osteoclast numbers in cultures with RA and OA synovial fluid. Panel (a) shows the mean osteoclast count in cultures with synovial fluid from RA (*n* = 13) and OA (*n* = 12) patients with healthy control serums as control. Both RA and OA synovial fluids increased the number of osteoclasts when compared to healthy serum. Surprisingly, OA synovial fluid increased the number of osteoclasts significantly more than RA synovial fluid. A difference in osteoclast phenotype was seen between cultures with RA (b) and OA (c) synovial fluids. 10x magnification confocal microscope images. The cells cultured with OA synovial fluid, which contained less inflammatory cytokines, were larger and contained more nuclei (*p* < 0.05) than the osteoclasts cultures with RA synovial fluid that contained higher concentrations of inflammatory cytokines. The osteoclasts cultured with healthy control serum (d) were even smaller and with fewer nuclei than in either culture with synovial fluid. Red color indicates TRAcP and blue Hoechst stain marks the nuclei. Changes in cell morphology are described in detail in Table 3.

Osteoclast numbers from the synovial fluid experiment are presented in Figure 4(a). Both RA (37.4 ± 8.50) and OA (57.0 ± 12.9) synovial fluids increased the number of osteoclasts significantly when compared to the culture with healthy serum (6.69 ± 6.44). Three OA and two RA samples were excluded due to technical issues in the samples. OA synovial fluid increased the osteoclast number significantly more than RA synovial fluid. The serum and synovial fluid cultures cannot be directly compared with each other because the mononuclear cells were collected from the donor at two different time points to limit the amount of donated blood to minimum and safe amounts. Also, the amount of used healthy serum was different between the experiments (20% in serum and 10% in synovial fluid experiment). In synovial fluid experiments, the healthy serum was used as a positive control, since in the earlier serum experiments it was found suitable. Thus, the use of FBS was avoided to save cells for the synovial fluid experiments.

Table 3 describes the cell morphology from the synovial fluid assay; the cells were analyzed visually independently and blindly by two researchers. Both RA and OA synovial fluid increased the number of nuclei in the osteoclasts significantly when compared to healthy serum; however, OA synovial fluid increased the nuclei count significantly more than RA. The cells cultured with OA synovial fluid were irregularly shaped and larger when compared to the ones cultured in the presence of RA synovial fluid. The intensity of TRAP staining in osteoclasts from synovial fluid cultures was weaker than in control samples. The number of stromal cells was also increased in the cultures with either synovial fluid. The interobserver reliability for osteoclast size (number of nuclei) was assessed by Cohen's kappa, which indicated strong agreement, *κ* = 0.803, *p* > 0.001. Example images of stromal cell scoring are shown in supplement (S4). Figures 4(b)–4(d) show confocal microscope images of osteoclasts from cultures with RA and OA synovial fluids and healthy serum.

### 3.3. Analysis of Bone Resorption

**Figure 5:**
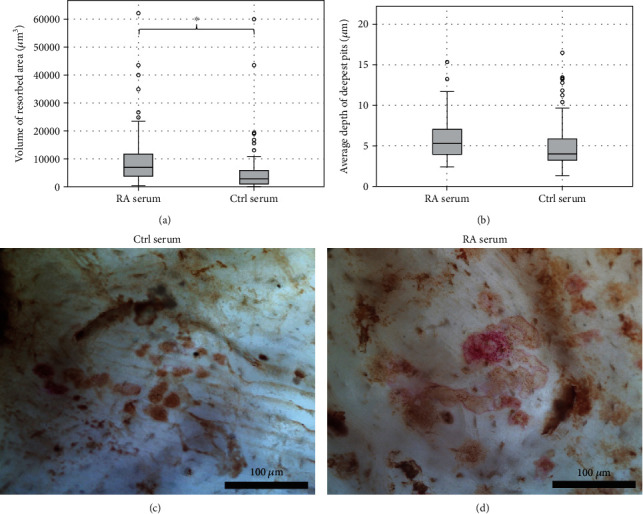
Bone resorption in cultures with RA and healthy serum. Panel (a) shows the average resorbed bone volume measured with a laser microscope from the serum experiment. Panel (b) shows the average depth of the deepest pits on the bone slices. Panels (c) and (d) show examples of WGA-lectin-stained resorption pits (brown color) on the bone slices. 40x magnification. In image (c), there is a single TRAcP positive cell remaining attached to the bone after the mechanical removal of cells by brushing.

**Figure 6:**
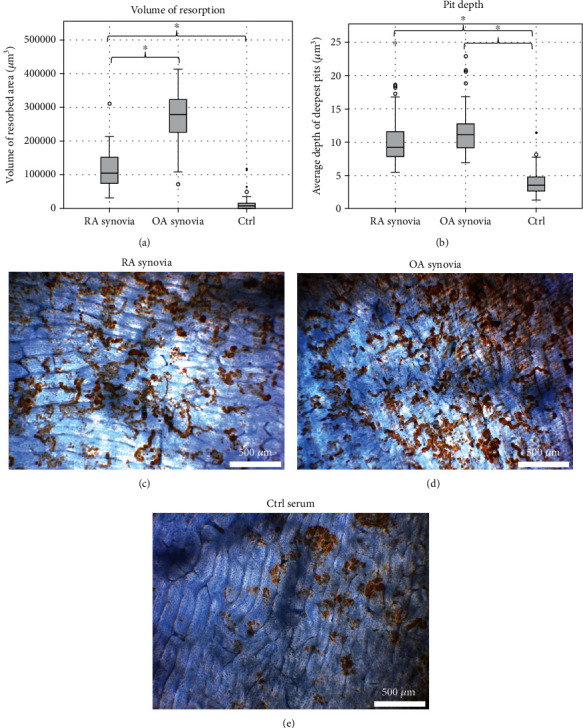
Bone resorption in cultures with RA and OA synovial fluid. Panel (a) shows the average resorbed bone volume measured with a laser microscope from the synovial fluid experiment. Example WGA-lectin stains of resorption pits on the bone slices from the cultures using RA and OA synovial fluids and healthy serum are shown in panels (c–e). 4x magnification. It was visually evident that the bone slices from the cultures with OA synovial fluid showed the most resorption.

WGA-lectin stain and light microscopy were used to confirm that resorption lacunae were present on the bone slices in both serum and synovial fluid tests. Example images of WGA-lectin-stained resorption pits on bone slices from the serum and synovial fluid experiments are shown in Figures 5(c) and 5(d) and 6(c)–6(e). A laser microscope was used to analyze the area and volume of resorption and average depth of the three deepest pits on the bone slices. Osteoclasts cultured with FBS showed resorption as a positive control.

The volume of resorption from the serum experiment is shown in Figure 5(a). RA serum increased both the area (14628 ± 12763 *μ*m^2^ vs. 5266 ± 4375 *μ*m^2^) and the volume (10368 ± 10695 *μ*m^3^ vs. 6048 ± 10157 *μ*m^3^) of resorption compared to healthy control serum (*p* < 0.05). The increase in resorption was in the same proportion as the increase in osteoclast number between the serums. No significant change was found in the average depth of the deepest pits between RA and Ctrl serum, Figure 5(b), as the small difference seen is within the reliable measurement accuracy.

The volume of resorption from the synovial fluid experiment is shown in Figure 6(a). Both RA (area: 67257 ± 21369 *μ*m^2^, volume: 115703 ± 52299 *μ*m^3^) and OA (area: 132961 ± 21611 *μ*m^2^, volume: 292121 ± 58025 *μ*m^3^) synovial fluids increased resorption of the bone slices, when compared to healthy control human serum (area: 24303 ± 24843 *μ*m^2^, volume 13329 ± 20014 *μ*m^3^) (*p* < 0.05). OA synovial fluid increased the area and volume of resorption, but the change in the average depth of the deepest pits between RA and OA was within measurement accuracy (Figure 6(b)). The synovial fluids increased the depth of the deepest pits when compared to the Ctrl serum. The depth of the pits with the control serum in the synovial fluid assay was the same as with the healthy control serums in the RA serum assay. A significant difference between the average depth of the deepest pits between healthy serum and all synovial fluid samples was seen (*p* < 0.05). The increases in resorption were in the same proportion as the changes in osteoclast numbers. A single osteoclast's resorption capacity was the same between different groups.

## 4. Discussion

The aim of this study was to investigate *in vitro* the effect of inflammatory factors present in RA serum and RA and OA synovial fluids on osteoclastogenesis and bone resorption by osteoclasts. The main finding emerging from our experiments was that, when compared to healthy controls, the inflammatory stimulus present in novel untreated RA patients' serum significantly increases the general osteoclastogenesis and bone resorption. A similar effect is seen in real life contributing to the secondary osteoporosis in RA patients [5, 22]. Since in RA the hypertrophic inflamed synovial tissue is the main source of RANKL and other inflammatory cytokines [18], the erosions tend to begin in the perichondral areas where the synovial tissue is in contact with the bone not covered by articular cartilage. In some RA patients, the joint decay can occur very fast. The bone erosions are caused by osteoclasts [23]. It is unlikely that the synovial cells are able to resorb bone as they exhibit a more macrophage- than an osteoclast-like phenotype, as they are TRAcP 5A not 5B positive [24]. Our data suggest that the systemic cytokines could contribute to enhanced bone resorption in RA, so we next wanted to study in closer detail the local environment adjacent to joints using synovial fluid preparations.

Previous studies have examined the increased proinflammatory cytokine profile in RA and OA synovial fluid and serum, and increased cytokine levels have been found to correlate with disease severity [25–27]. Our cytokine assay data (Table 2, Figure 2 and supplementary file S1) is in accordance with the previous literature. Our whole cytokine data (Supplementary file S1) showed a connection between synovial fluid OPG and the patient KL score. In earlier studies, elevated levels of OPG expression have been shown in damaged osteoarthritic cartilage [28]. In our synovial fluid and serum samples, the levels of major proinflammatory cytokines are increased with the greatest increases in cytokines associated with RA, as expected, such as IL-8, IL-6, IL-17, and VEGF. The measured cytokines were roughly ten times more concentrated in synovial fluid than in serum. The proinflammatory cytokines in synovial fluid are generally thought to be locally produced in the inflamed synovial tissue by inflammatory cells and filtrated into serum. We see that the results of this study represent the effects of the complex interconnected cytokine networks found in different conditions. Below, we go through in more detail how these networks explain our current findings.

RANKL is considered the main factor of osteoclast differentiation [29]. To verify our assay, complete deprivation of RANKL in the negative control samples inhibited osteoclastogenesis also in our study; hence, our data agrees that RANKL signaling is the most crucial element for osteoclastogenesis. To evaluate the effect of other cytokines and chemokines present in the tested patient samples, supraphysiological RANKL concentrations were used to normalize the involvement of the natural RANKL on osteoclast differentiation. Various cytokines that promote osteoclastogenesis via RANKL or another pathway are relatively well known already.

Cytokines associated with increased osteoclastogenesis include TNF-*α*, IL-1, IL-6, IL-7, IL-8, IL-15, and IL-17 [11] that were all found in increased concentrations in the RA SF samples compared to OA. TNF-*α*, IL-6, IL-7, and IL-17 were also increased in RA serum compared to healthy controls. Along with the above cytokines, various other disease-related molecules and proteins such as CRP, VEGF, IL-11, IL-23, and IL-34 in RA are also known to increase osteoclastogenesis independently of RANKL [11, 30, 31]. Others were not tested, but a notable increase of VEGF was seen in the tested RA synovial fluid samples.

We could see the pro-osteoclastogenic effect of RA serum in the osteoclast cultures as increased number of osteoclasts (Figure 3), number of pits, and as resorbed area (Figure 5) in comparison to healthy controls. A higher total amount of cytokines in synovial fluid compared to sera increased the osteoclastogenesis even further. The main likely explanatory factors for the increase are IL-6 and IL-8 that are elevated both in OA and RA synovial fluid (Table 2). IL-6 has been shown to increase the number of osteoclasts [32] in addition to this IL-8 both stimulates osteoclastogenesis and increases their resorption activity [33, 34]. Interestingly, the higher total amount of proinflammatory cytokines in RA synovial fluid samples, when compared to OA synovial fluid, did not directly lead to stronger osteoclast differentiation or higher resorption activity. This could be due to osteoclastogenesis-inhibiting factors. We found increased concentrations of IL-10 in our RA serum samples compared to healthy controls, and IFN-*γ*, IL-4, and IL-10 in RA synovial fluid samples compared to OA. Even though these results should be interpreted with caution due to low number of samples and shown RF interference in IL-4 and IL-10 measurements, these have all been shown to decrease osteoclastogenesis under some conditions [11]. IL-4, IL-13, and IL-10 all drive monocytes to M2 macrophages [35]; thus, the complex pro and antiosteoclastogenic effects drive the differentiation and activity simultaneously. Interestingly, earlier studies have shown increased concentrations of also other osteoclastogenesis-inhibiting factors, INF-*α*, IL-3, IL-27, and IL-33 in RA patient samples [36–39]. Together, these findings could explain some of the differences in RA and OA bone loss. Because of the limited number of samples in our study, and the serum and synovial fluid cultures being done at two different time points, we see that our results should be viewed at a general level. Further studies are needed on the detailed signaling events of individual cytokines.

We found evidence of morphological differences of osteoclasts that were generated under RA and OA synovial fluids and believe that this change in the phenotypes of these cells is due to inflammatory osteoclastogenesis [2]. The osteoclasts cultured with OA synovial fluid were larger and contained more nuclei than those cultured with RA synovial fluid. It has been suggested that in inflammatory osteoclastogenesis, the proinflammatory cytokines cause increased osteoclastogenesis, but cause the osteoclasts to remain in a more macrophage-like phenotype with fewer nuclei and smaller size [2, 18]. This in turn will affect the regulation of bone resorption and communication with other inflammatory cells. Interestingly, when the resorption pit volumes were analyzed, the change in cell morphology did not affect the area resorbed by a single osteoclast, as the changes in resorbed bone volume were a direct result of the increased number of osteoclasts. The cell cultures were done using supraphysiological RANKL levels to evaluate the effect of other inflammatory cytokines, and under these circumstances, high levels of monocyte differentiation regulating cytokines resulted in decreased osteoclastogenesis in samples with RA synovial fluid compared to OA synovial fluid with less inhibitory factors.

As limitations of our study, we acknowledge, that both RA and OA are very variable diseases, and we see this variation in cytokine levels between the patients. Even though our experiments were performed on cells from a single donor, we would expect the results to be similar with slight variations if cells from a different healthy donor were used. However, we must be careful in the interpretation of this data. Especially, cells derived from RA or OA patients could give a different response due to the priming in inflammatory milieu or genetic factors. The donor used in this study does not have any rheumatic disease or osteoarthritis. In further studies, also different donors for osteoclast precursors should be considered. However, this study shows an example of how the local RA or OA inflammatory environment can change the behavior of healthy individual's cells.

Our study would have benefitted from treatment-naïve OA controls for RA serum samples and healthy SF controls for the end-stage RA knees at time of a prosthesis operation. This was governed by the availability of patient material for OA and due to ethical reasons for the healthy controls. OA diagnosis is mostly done in primary healthcare, which data we do not have access to, years before needing treatment in specialized healthcare. Also determining what would classify as a treatment-naïve OA patient would be extremely hard as the patients seek medical help at very different stages of the disease, as the symptoms do not often correlate with the radiological findings, and the first line of treatment is nonsteroidal anti-inflammatory medication, which can be bought over the counter in pharmacies. The OA patients going through operative treatment are a less heterogenous selected group. The low sample volumes forced us to pool synovial fluid samples for the cell cultures, which we also consider as a limitation of our study.

## 5. Conclusion

In this preliminary study, we showed how local and systemic inflammatory cytokines in RA have a direct effect on the differentiation and bone resorption ability of osteoclasts *in vitro*. RA serum and synovial fluid both increased osteoclast differentiation and bone resorption capacity, when compared to healthy serum. Osteoclast differentiation and bone resorption increased even more in the presence of OA synovial fluid that could be due to lower levels of monocyte differentiation regulating cytokines. These data help us to better understand these diseases and remind us how complex the inflammatory processes are. Further studies of inflammatory osteoclastogenesis are required to obtain an understanding for optimal therapeutic interventions.

## Figures and Tables

**Table 1:** Patient data for samples used in cell experiments.

	Age (years) ± SD	Gender	Mean KL knee score (range)
RA synovial fluid	61.3 ± 7.2	2 females 1 male	3 (2-4)
OA synovial fluid	58.0 ± 4.4	2 females 1 male	3.3 (3-4)
RA serum	47.0 ± 5.2	2 females 1 male	
Ctrl serum	28.7 ± 2.5	2 females 1 male	

The mean age, gender, and Kellgren-Lawrence knee score of patients and controls at the time of sample acquisition. Range refers to the difference between the lowest and highest score.

**Table 2:** Cytokine concentrations (pg/ml) of serum and synovial fluid samples used for osteoclastogenesis experiments.

	RA serum		Control serum		RA SF		OA SF	
	Mean	SD	Mean	SD	Mean	SD	Mean	SD
OPG					423	329	400	325
sRANKL					<7	—	<7	—
MIP-1b	30	1	30	12	934	1515	30	20
IL-6	7	2	<2	—	1365	1850	123	121
IFN-y	23	6	25	14	133	134	7	6
IL-1ra	37	7	5	9	2169	3304	14	24
IL-5	2	2	4	6	5	6	<2	—
GM-CSf	<3	—	<3	—	36	25	<3	—
TNF-*α*	34	20	28	13	95	83	4	7
RANTES	988	62	396	84	132	91	32	48
IL-2	<1	—	<1	—	7	8	<1	—
IL-1b	1	1	1	1	16	17	<1	—
Eotaxin	55	17	62	27	42	35	8	4
Basic FGF	5	5	5	5	19	18	7	4
VEGF	21	24	2	2	1066	996	346	224
PDGF-BB	1049	413	910	522	26	26	1	2
IP-10	387	114	190	52	16342	22064	332	118
IL-13	3	1	2	1	14	12	4	2
IL-4	2	0	2	0	3	3	<1	—
MCP-1	<12	4	<12	—	98	72	52	23
IL-8	<4	—	<4	—	3775	3793	19	29
MIP-1a	2	2	2	1	115	184	4	4
IL-10	6	1	<5	—	40	37	<5	—
G-CSF	40	18	27	8	77	52	14	7
IL-15	<6	—	<6	—	26	24	<6	—
IL-7	8	3	6	1	27	35	3	2
IL-12(p70)	15	5	2	2	110	86	37	16
IL-17	97	33	74	17	61	90	0	0
IL-9	58	56	18	3	28	34	<2	1

**Table 3:** Cell morphology in synovial fluid assay.

	TRAcP intensity	Number of nuclei	Stromal cells
**RA** (*n* = 13)	2	4-7^∗^	2^∗^
**OA** (*n* = 12)	2	>8^∗^/^∗∗^	2^∗^
**Ctrl** (*n* = 15)	3	2-3	1

Each bone slice was analyzed blindly and independently by two researchers. TRAcP intensity and the presence of stromal cells were analyzed on a 0-3 scale and the average number of nuclei per cell was counted. ^∗^/^∗∗^*Fisher's exact test,p* < 0.05. Example images are shown in Figure 4 and S4.

## Data Availability

The data used to support the findings of this study are included within the article and the supplementary materials.

## Abbreviations

ACPAs: Anticitrullinated protein antibodies
ACR/EULAR: American College of Rheumatology/European League Against Rheumatism
Ctrl: Control
DAB: 3,3′-Diaminobenzidine
FBS: Fetal bovine serum
FGF: Fibroblast growth factor
G-CSF: Granulocyte colony-stimulating factor
GM-CSf: Granulocyte-macrophage colony-stimulating factor
IFN: Interferon
IL: Interleukin
KL knee score: Kellgren-Lawrence knee score
MCP-1: Monocyte chemoattractant protein 1
M-CSF: Macrophage colony-stimulating factor
OA: Osteoarthritis
OPG: Osteoprotegerin
OPN: Osteopontin
PBS: Phosphate-buffered saline
PDGF-BB: Platelet-derived growth factor
PFA: Paraformaldehyde
RA: Rheumatoid arthritis
RF: Rheumatoid factor
RANKL: Nuclear factor kappa-*Β* ligand
RANTES: Regulated upon Activation, Normal T Cell Expressed and Presumably Secreted
SD: Standard deviation
SF: Synovial fluid
WGA: Wheat germ agglutinin.
TNF-*α*: Tumor necrosis factor-alpha
TRAcP/TRAP: Tartrate-resistant acid phosphatase
VEGF: Vascular endothelial growth factor
WGA: Wheat germ agglutinin.

## Supplementary Materials

Supplementary 1 S1 table; cytokine data from serum and synovial fluid samples.

Supplementary 2 S2 table: correlations between cytokines within each group of samples.

Supplementary 3 S3 figure: number of osteoclasts in cultures with fetal bovine serum.

Supplementary 4 S4 document: example images of stromal cell scoring from synovial fluid experiments.
