# Supplementary material for: Preliminary Report: Osteoarthritis and Rheumatoid Arthritis Synovial Fluid Increased Osteoclastogenesis In Vitro by Monocyte Differentiation Pathway Regulating Cytokines
Source: Mediators Inflamm. 2022 May 31;2022:2606916. doi: 10.1155/2022/2606916 (PMC9175097; doi:10.1155/2022/2606916)
Supplement: Supplementary 3 — S3 figure: number of osteoclasts in cultures with fetal bovine serum. [file 2606916.f3.pptx]

## Slide 1
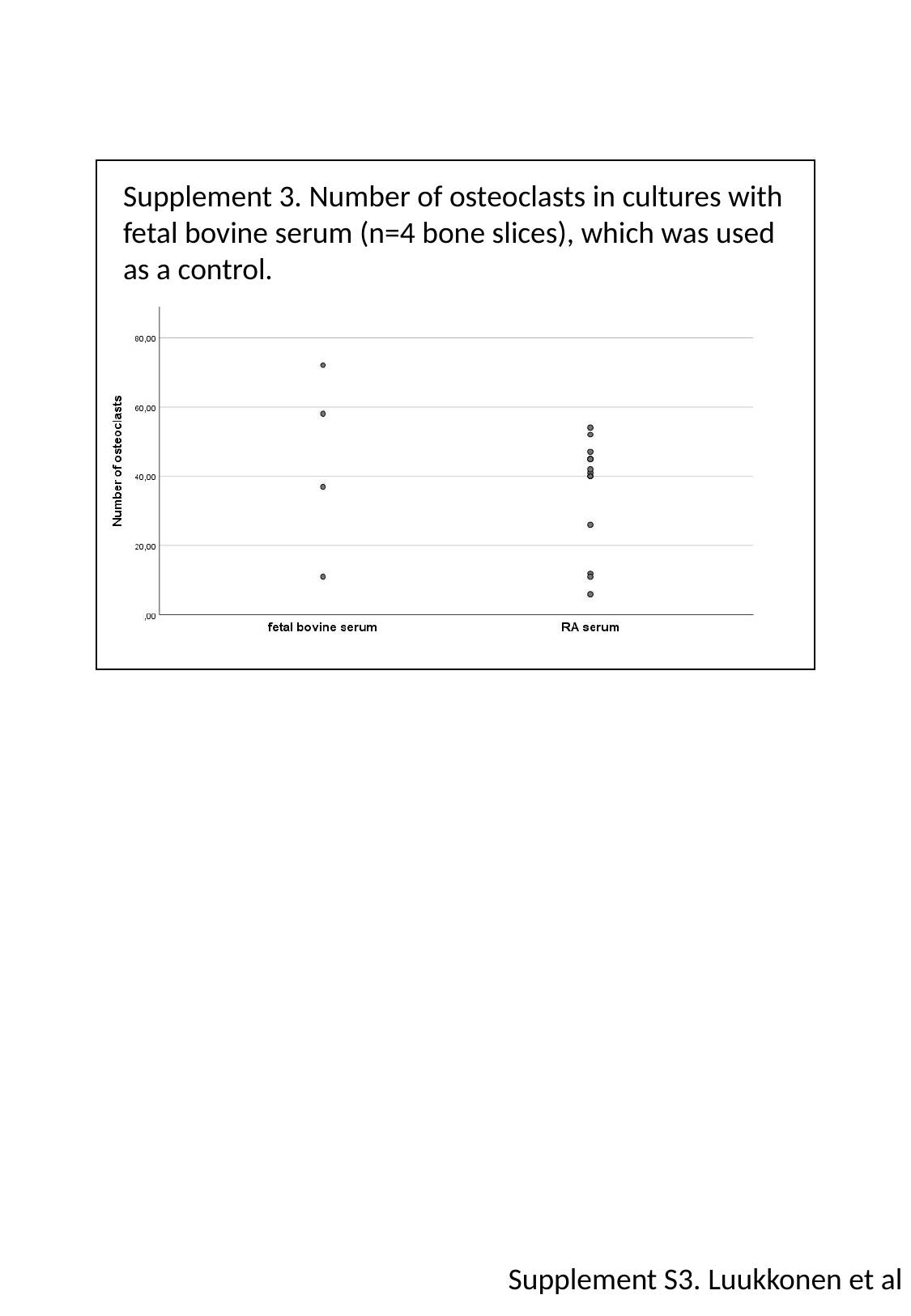

Supplement 3. Number of osteoclasts in cultures with fetal bovine serum (n=4 bone slices), which was used as a control.
Supplement S3. Luukkonen et al
