# Supplementary figures and images for: Preliminary Report: Osteoarthritis and Rheumatoid Arthritis Synovial Fluid Increased Osteoclastogenesis In Vitro by Monocyte Differentiation Pathway Regulating Cytokines
Source: Mediators Inflamm. 2022 May 31;2022:2606916. doi: 10.1155/2022/2606916 (PMC9175097; doi:10.1155/2022/2606916)

**Supplement 4. Examples of stromal cell scoring on +, ++, +++ scale.**

+


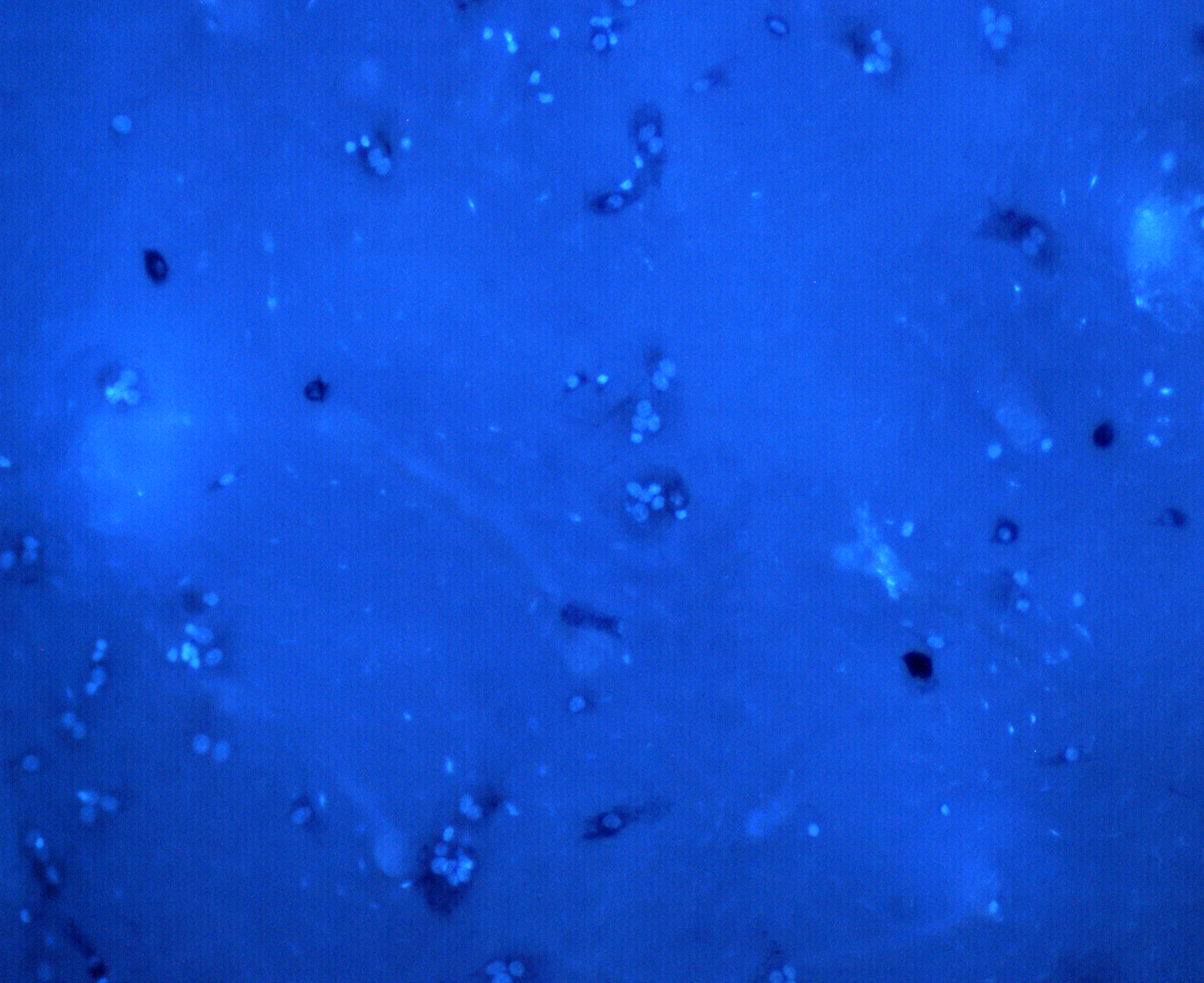


++


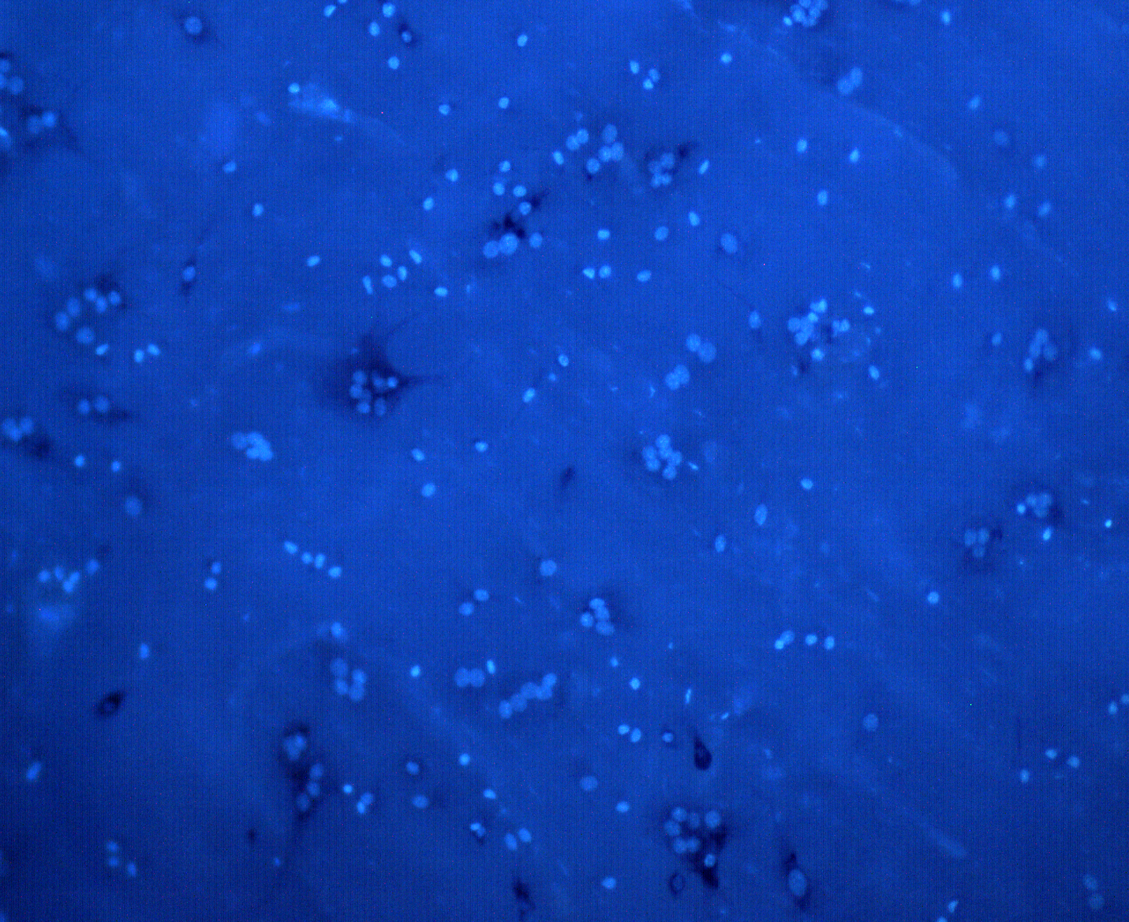

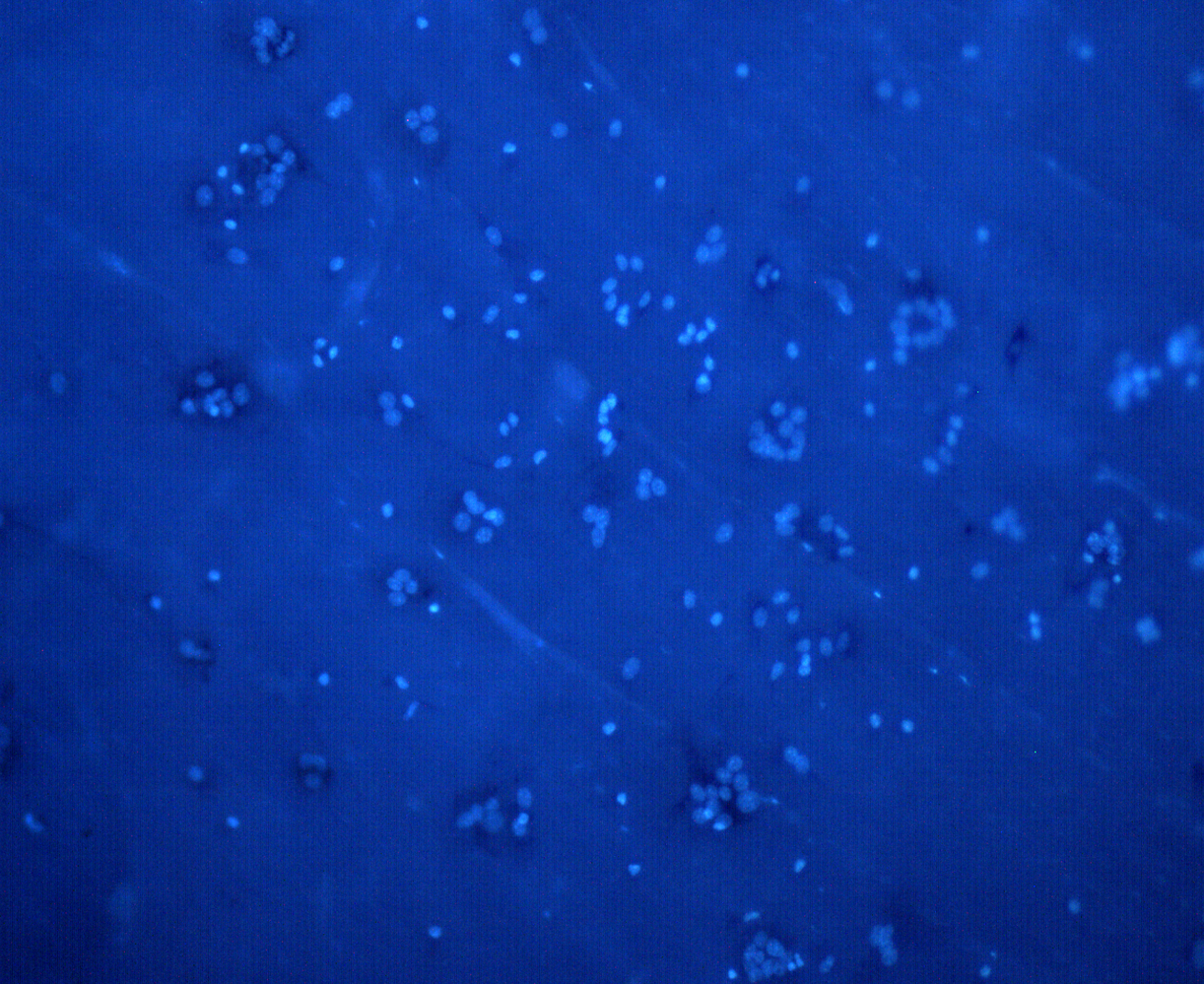


+++


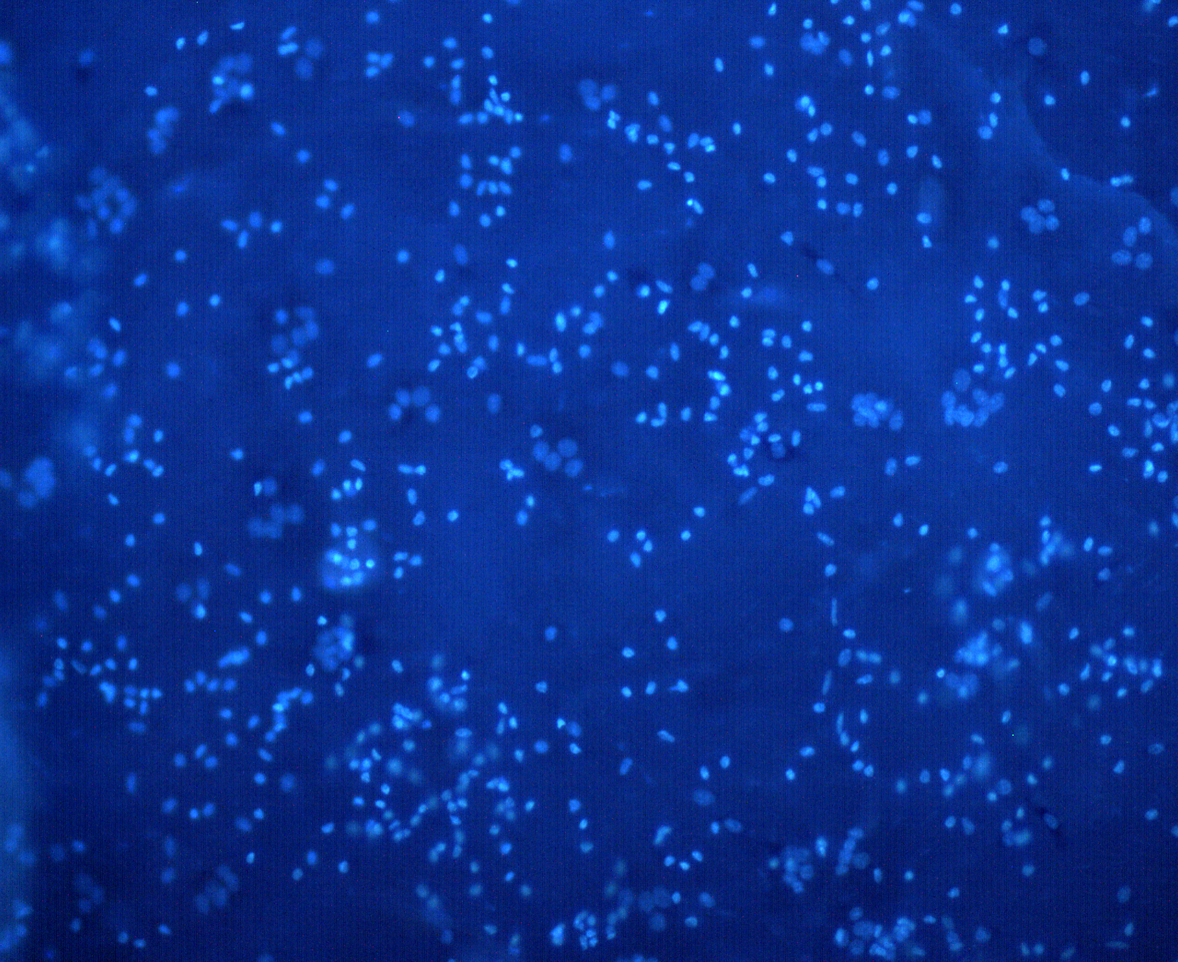

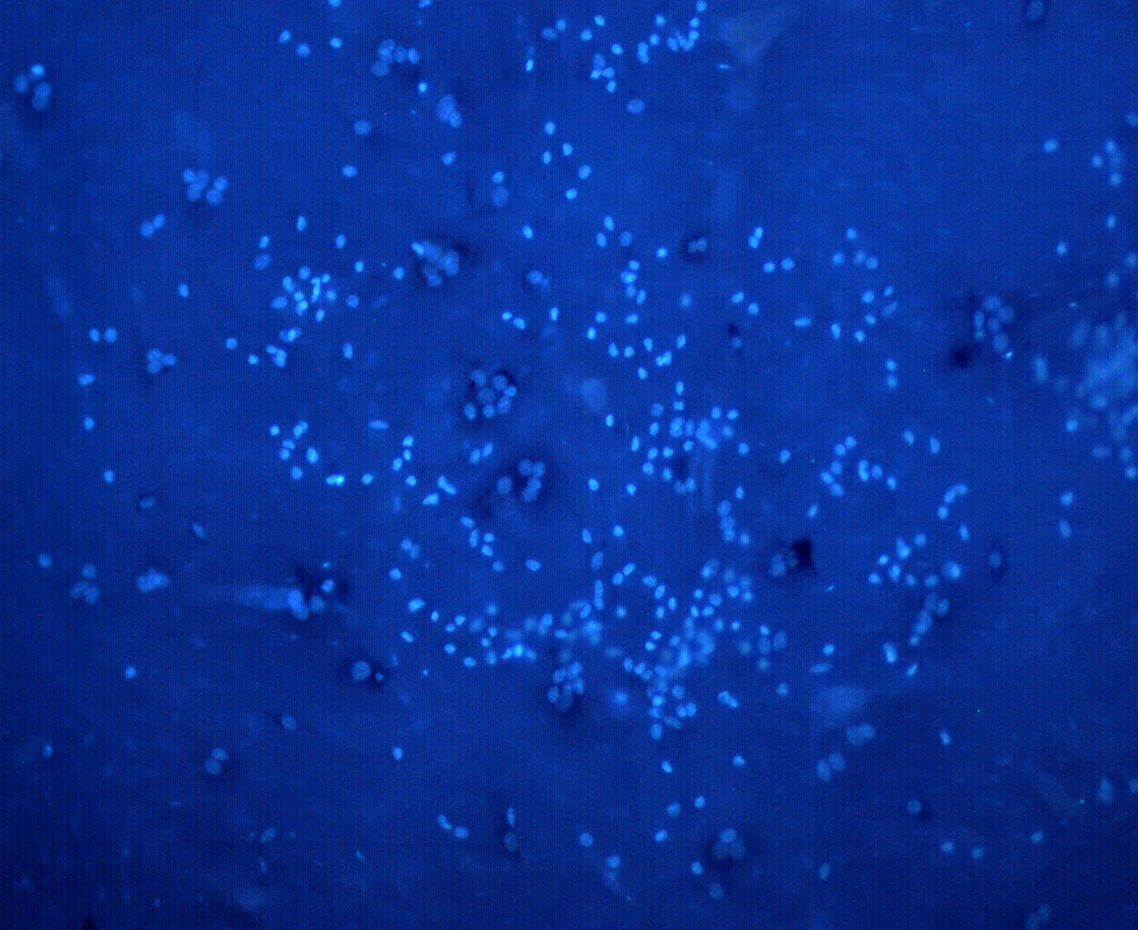

Supplement: Supplementary 4 — S4 document: example images of stromal cell scoring from synovial fluid experiments. [file 2606916.f4.docx]
